# Prevention and Treatment of Postmastectomy Lymphedema: A Physiotherapy Perspective

**DOI:** 10.3390/curroncol32100555

**Published:** 2025-10-03

**Authors:** Rosalba León-Díaz, Andrea Medina-Otero

**Affiliations:** 1 Laboratorio de Biología y Salud Integral, Instituto de Investigaciones Biológicas, Universidad Veracruzana, Xalapa-Enríquez 91190, Mexico; 2 PhysiOne Xalapa, Xalapa-Enríquez 91020, Mexico; physionxalapa@gmail.com

**Keywords:** lymphedema, mastectomy, lymphadenectomy prevention, lymphokinetic exercises, physiotherapy, breast cancer, risk factor, lymphatic system

## Abstract

Lymphedema is a frequent complication associated with breast cancer treatment. It is estimated that up to 30% of patients undergoing mastectomy develop this condition within 12 to 24 months post-surgery. In Mexico, the limited emphasis placed on lymphedema prevention in breast cancer patients is reflected in the insufficient coverage of this issue within official medical guidelines. In this review, research articles, systematic reviews, and official treatment guidelines were retrieved from PubMed, Google Scholar, Elsevier, SciELO, and Redalyc databases, to examine studies about the application and effectiveness of physiotherapy techniques for the prevention, diagnosis, and treatment of postmastectomy lymphedema. Our findings indicate that complex decongestive therapy (CDT) is considered the first-line treatment for lymphedema. Among its components, compression therapy shows the strongest individual evidence base. Nevertheless, studies consistently demonstrate that the combined use of all four components of CDT (manual lymphatic drainage, compression, skin care, and exercise) results in superior patient outcomes. Despite this, CDT is not routinely implemented as a standard of care for patients following mastectomy and/or lymphadenectomy in Mexico. Therefore, there is a pressing need to promote the inclusion of physiotherapy strategies, particularly CDT, in the prevention and management of postmastectomy lymphedema within national healthcare protocols.

## Simple Summary

In Mexico, many breast cancer patients face a high risk of lymphedema, a chronic swelling caused by surgery or radiotherapy. Although national guidelines mention rehabilitation, they give little detail on how to prevent or treat it, leaving patients and professionals with limited guidance. About 30% of women undergoing surgery develop this condition, often within the first year, and obesity or late-stage diagnosis increases the risk. Studies show that supervised exercise, early prevention programs, and Complex Decongestive Therapy improve outcomes. Expanding training for health professionals and integrating surveillance models could guide public health policy and help preserve quality of life for Mexican women.

## 1. Introduction

Lymphedema is one of the most frequent and disabling sequelae of breast cancer treatment. It results from the interruption of lymphatic pathways, particularly during axillary dissection, which leads to the accumulation of lymphatic fluid. Clinically, lymphedema manifests as chronic and progressive swelling of the operated arm, accompanied by heaviness, pain, restricted mobility, and a substantial psychological and social burden for patients [1].

It is estimated that up to 30% of mastectomy patients develop lymphedema within 12 to 24 months after surgery. In Mexico, however, awareness of lymphedema prevention among breast cancer patients remains insufficient, as reflected in the limited attention devoted to this condition in official medical guidelines. Patients should be adequately informed about risk factors, early identification of symptoms, and the importance of timely rehabilitation interventions. These elements are essential for secondary prevention, since their omission or lack of awareness may lead to serious complications, including recurrent infections, elephantiasis (advanced-stage lymphedema), and, in rare cases, lymphangiosarcoma [2].

Despite the availability of effective physiotherapeutic approaches, such as complex decongestive therapy (CDT), these interventions are not consistently integrated into routine clinical practice in Mexico. Among the components of CDT, compression therapy has the strongest evidence when applied individually; however, studies consistently demonstrate that the combined use of manual lymphatic drainage, compression, skin care, and exercise yields superior patient outcomes.

Given this context, the information provided to breast cancer patients regarding the etiology, clinical manifestations, and management of lymphedema remains limited within the Mexican healthcare system. For that reason we prepare this narrative review to underscore the importance of preventive strategies aimed at reducing the incidence and severity of postmastectomy lymphedema.

## 2. Methods

We retrieved (from 1990 to 2024) research articles, systematic reviews, and official guidelines from PubMed, Google Scholar, Elsevier, SciELO, and Redalyc databases to analyze the available evidence on the prevention and treatment of lymphedema, with particular emphasis on the role of physiotherapy.

## 3. Lymphedema: An Underlying Issue

### The Lymphatic System: Functions and Significance

The lymphatic system, together with the venous and arterial networks, constitutes a key component of the circulatory system. Its primary functions are to collect and transport lymph and tissue fluids, and to return them to the bloodstream. It plays a crucial role in maintaining interstitial homeostasis by absorbing fluid that has extravasated from blood vessels into the interstitial space and reintegrating it into the general circulation [3].

Anatomically, the lymphatic system is composed of lymphatic vessels, lymph nodes, and lymph. According to Ciucci, Vadra, and Sorocco [4], the lymphatic vessels of the upper limb (arm and forearm) originate at the capillary level of the microcirculation and are divided into superficial and deep networks. Lymph—the fluid transported through these vessels—eventually drains into the subclavian veins, merging with the blood circulation. It is formed when interstitial fluid enters the initial lymphatic capillaries, passes through pre-nodal collectors, and flows into the lymph nodes, where it undergoes filtration. From there, it exits through post-nodal collectors and continues through deeper lymphatic vessels.

## 4. Lymphedema Pathophysiology: Essential Concepts

Leduc [3] describes lymph formation based on Starling’s hypothesis, which explains the balance between filtration and reabsorption processes at the capillary level. Water enriched with nutrients, minerals, and vitamins leaves the lumen of arterial capillaries and diffuses into the interstitial space, where it nourishes the cells. Metabolic waste products are then released back into this interstitial fluid. A portion of this fluid is reabsorbed into the venous capillaries, a process determined by two opposing pressures: (1) hydrostatic pressure, driven by cardiac activity, is approximately +30 mmHg at the arterial capillary end and decreases to about +20 mmHg at the venous capillary end, and (2) oncotic pressure, generated by plasma proteins, is about –25 mmHg.

The equilibrium between these forces ensures fluid balance: at the arterial end, the predominance of hydrostatic pressure promotes filtration into the interstitial space, while at the venous end, the oncotic gradient favors reabsorption. Any disruption of this delicate balance can contribute to impaired lymphatic drainage and the subsequent development of lymphedema. An imbalance between capillary filtration and reabsorption results in excess interstitial fluid, which is normally absorbed by a network of lymphatic capillaries. These capillaries collect fluid and metabolic waste, transport them through lymphatic collectors, and deliver them to lymph nodes, which in turn return lymph to the venous system for subsequent elimination [3].

Clinically, lymphedema becomes apparent when the accumulated interstitial lymph volume exceeds approximately 20% of the normal arm volume. This condition may arise through two mechanisms: (1) dynamic insufficiency, in which excessive lymph production surpasses the absorptive and compensatory capacity of the lymphatic system, leading to edema; and (2) mechanical insufficiency, caused by primary or secondary disruption of lymphatic pathways, resulting in impaired lymph transport [5].

Fluid transport through the lymphatic system is essential for lipid homeostasis, immune function, and tissue fluid balance. Lymphedema is therefore considered the result of a chronic inflammatory process driven by the lymphatic system’s inability to adequately absorb and transport interstitial fluid. This process involves several molecular mechanisms, including gene-mediated regulation of inflammatory responses. Experimental models have shown that lymph node removal can cause the rupture of collecting vessels, the accumulation of fluid in the dermis, and the redirection of lymphatic drainage into collateral vessels [5].

Hull [6] reported that damage to the lymphatic system disrupts lymph flow, leading to fluid accumulation, vessel distension, valvular dysfunction, and reflux. These alterations contribute to tissue impairment, remodeling, lymphatic hyperplasia, adipocyte deposition, and fibrosis, culminating in the clinical manifestation of lymphedema.

Findings from secondary lymphedema in a murine tail model demonstrated pathological changes after surgery, including chronic edema, proliferation of subcutaneous adipose tissue, fibrotic deposition, progressive lymphocyte infiltration, and lymphatic hyperplasia. While most inflammation-related symptoms subsided within eight weeks, fibrosis persisted, underscoring the irreversible nature of this process and the critical importance of early therapeutic intervention to prevent lymphedema progression [7].

## 5. Diagnosis

Early and accurate diagnosis is essential for establishing an effective treatment plan. In most patients, lymphedema can be diagnosed based on medical history and physical examination. Some authors consider lymphoscintigraphy the gold standard. This technique involves the injection of a radiolabeled protein (^99m^Tc-sulfur colloid), which emits gamma rays detected by a specialized camera. It allows for the identification of delayed transit, protein accumulation, asymmetric uptake, and the formation of collateral lymphatic channels [8]. However, this method is not routinely available in Mexico.

Traditionally, perimetric measurements with a tape measure have been the preferred clinical diagnostic method. Measurements are taken at 4 cm intervals from the ulnar styloid to the axillary fold. A difference of ≥2 cm between the affected and contralateral limb is considered diagnostic. However, this technique has limited sensitivity for detecting subtle changes [9,10,11].

More reliable information on lymphedema severity and progression is obtained using volumetric assessment techniques, including the water displacement method, perometry, dual-energy X-ray absorptiometry, magnetic resonance imaging, computed tomography, and three-dimensional scanning [10].

Another non-invasive method is bioimpedance spectroscopy, which estimates extracellular fluid content by measuring tissue resistance to an applied electrical current. Increased interstitial fluid corresponds to decreased resistance [12]. A South Korean study of 29 patients demonstrated a strong correlation between bioimpedance and clinical measurements (perimetry and volumetry), showing that bioimpedance is more sensitive to subtle changes and may be particularly useful for long-term monitoring after complex decongestive therapy [13].

Tonometry is also employed to assess tissue resistance, detecting the presence of fluid and fibrosis. However, its reliability is debated: while Moseley and Piller [14] reported its utility, they found it less effective than bioimpedance. Another advanced method is infrared lymphofluoroscopy, which uses indocyanine green (a non-radioactive contrast agent) that fluoresces under infrared light. This technique allows for the real-time visualization of lymphatic pathways and can help identify functional channels in postmastectomy patients, guiding therapeutic interventions such as lymphatic drainage [15].

Despite their value, high-technology diagnostic techniques such as bioimpedance, tonometry, and lymphofluoroscopy remain costly and inaccessible for many patients and health services in Mexico. Moreover, evidence does not consistently show superior diagnostic accuracy compared with traditional clinical assessment. Importantly, the first detection of lymphedema often comes from patients themselves, based on the recognition of early symptoms and localized swelling [16].

According to the International Society of Lymphology (ISL), lymphedema can be staged as follows [17]:

- Stage 0 (Latency/Asymptomatic): no visible swelling, but patients may report tingling, heaviness, or fatigue in the limb.
- Stage 1 (Mild): early swelling due to protein-rich interstitial fluid. Pitting edema is present, but reversible with treatment or arm elevation; no permanent tissue damage has occurred.
- Stage 2 (Moderate): persistent swelling not relieved by elevation; pitting is absent. Tissue changes (inflammation, fibrosis, thickening) develop and are irreversible, although treatment can control progression.
- Stage 3 (Severe/Advanced): marked enlargement, fibrosis, and skin hardening. Elephantiasis may be observed in this stage.

## 6. Prevention

Prevention is one of the most important tools in any healthcare system. The World Health Organization (WHO) defines disease prevention as measures aimed not only at reducing the risk of onset, but also at halting progression and mitigating consequences once the condition has developed.

The quality of life of patients after mastectomy largely depends on measures taken to prevent treatment-related sequelae. For this reason, it is essential to provide patients with clear information on the risk of developing lymphedema and strategies for its prevention. This includes early recognition of symptoms, awareness of risk factors, adoption of self-care practices, and timely access to treatment. Table 1 provides a synthesis of the key evidence regarding strategies for lymphedema prevention.

Although lymphedema is a chronic and progressive condition with no definitive cure, its progression can be controlled when treatment begins at the first signs of onset, thereby preserving quality of life. Patients at risk should be educated about proper hygiene, care of the affected limb, and preventive measures that enable early detection and prompt management. Prevention begins at the surgical stage: less invasive techniques, such as sentinel lymph node biopsy, result in less damage to the operated limb compared to axillary lymph node dissection performed for pathological findings or when sentinel node biopsy is omitted [26,27].

Following surgery or radiotherapy, patients are advised to implement specific preventive measures to reduce risk factors. These include avoiding venipuncture and blood pressure measurements in the affected arm; avoiding acupuncture; maintaining daily skin hygiene with mild soaps and proper drying (especially folds and interdigital spaces); using emollients; and avoiding depilatory methods that may damage the skin, such as waxing or shaving with blades [17,26,27]. Occupational activities involving strenuous use of the affected limb are associated with more advanced clinical stages of lymphedema [28].

Evidence from international studies highlights the importance of patient education. A Spanish study with 65 patients found that only 24.6% received information after surgery, 37.5% had ever heard the term lymphedema, and just 3% were informed about risk factors [20]. Despite being dated, these findings underscore the continuing need for improved patient education, particularly in countries such as Mexico.

Interventional studies demonstrate the effectiveness of preventive education. At the University of Genoa, Italy, 55 women undergoing axillary lymph node dissection were divided into a control group and an intervention group that received preventive education. After 24 months, lymphedema developed in 33% of the control group, compared to only 8% of the intervention group [23]. Similarly, a study in Tamil Nadu, India, evaluated a preventive protocol in 120 mastectomy patients. Those receiving a comprehensive protocol (exercise, self-care, education in self-drainage, and compression sleeves) had a significantly lower incidence of lymphedema and improved quality of life compared with those who received routine care, regardless of age, BMI, or extent of axillary dissection [25].

Risk stratification tools also contribute to prevention. In Beijing, China, a scoring system was developed to predict lymphedema risk in 533 breast cancer patients (mean age 57 years). The checklist included six categories: type of surgery, axillary lymph node surgery, early postoperative edema, neoadjuvant chemotherapy, radiotherapy, and use of the affected arm for heavy lifting. Scores ranged from 8 to 22, with higher scores predicting greater risk. This tool allows early identification of high-risk patients, enabling closer monitoring, patient education, and timely referral to physiotherapy or specialized care [21].

Exercise plays a central role in prevention. A systematic review of 24 randomized controlled trials demonstrated that well-structured exercise programs, guided by trained professionals and initiated as early as seven days after surgery, improve shoulder mobility and functionality while effectively reducing lymphedema risk. The review found limited evidence supporting manual lymphatic drainage (MLD) alone, although one study combining MLD with exercise showed reduced risk, and another combining MLD with compression demonstrated benefits but had an insufficient sample size for conclusive results. Importantly, progressive exercise did not increase lymphedema risk compared with restricted activity [22].

Finally, Stout et al. [19] proposed a surveillance model for physical rehabilitation in breast cancer care, emphasizing three stages: preoperative, early postoperative, and surveillance. The model integrates patient education, reduction in risk factors, and early rehabilitation interventions, enabling the timely detection of functional impairments, including lymphedema. Implementation of this model not only prevents sequelae, but also fosters healthy behaviors during and after cancer treatment [19].

## 7. Treatment

Management of upper extremity lymphedema secondary to breast cancer surgery comprises two main modalities: conservative and surgical approaches [17,26]. Conservative treatment remains the cornerstone of care, primarily through physiotherapeutic techniques that reduce edema, alleviate pain, improve mobility, and ultimately enhance patients’ quality of life [29]. Table 2 provides an overview of the key treatment approaches and findings related to lymphedema.

In 2010, a clinical trial conducted by physicians and physiotherapists at the University of Alcalá (Spain) evaluated the effectiveness of early physiotherapy in preventing secondary lymphedema after breast cancer surgery. The study included 120 women who underwent axillary dissection, randomly assigned to either an intervention group (self-care education, manual lymphatic drainage, scar massage, and progressive shoulder exercises guided by a physiotherapist) or a control group (education only). After one year, lymphedema developed in 18 of the 116 women who completed follow-up: 14 in the control group and only 4 in the intervention group. These findings support early physiotherapy programs as significantly more effective than education alone in reducing the risk of secondary lymphedema [18,40].

The standard conservative protocol is complex decongestive therapy (CDT), a multimodal treatment developed in the 1980s by Dr. Michael Földi, building upon Vodder’s manual lymphatic drainage method and complemented with additional techniques. CDT comprises four essential components: (1) manual lymphatic drainage (MLD), (2) compression therapy (bandages or garments), (3) lymphokinetic exercises, and (4) skin care and risk factor prevention [3,34].

The objectives of CDT are to reduce limb circumference, minimize tissue fibrosis and infection risk, and improve mobility [3]. CDT is delivered in two phases: (a) intensive phase, focused on reducing edema through daily MLD, compression bandaging, exercises, and hygienic-dietary measures. This phase typically lasts 3–8 weeks. (b) Maintenance phase, aimed at preserving results, involving the continuous use of compression sleeves, self-care, and exercise routines supervised by the therapist. Suitability for CDT is determined according to each patient’s clinical history [15,16,17].

In Mexico, a recent study evaluated the effect of CDT in 32 patients with postmastectomy lymphedema, treated by the Asociación Mexicana de Linfología y Linfedema A.C. The intensive phase lasted five days and included patient education on prevention and self-care, followed by MLD using the Vodder–Földi technique. Multilayer short-stretch bandaging was applied, providing higher pressure during movement and lower pressure at rest. Bandages were worn continuously for 24 h and replaced in each session by trained personnel. Lymphokinetic exercises were also incorporated to stimulate the muscle pump and facilitate lymphatic flow. The results demonstrated significant reductions in limb volume and improvements in quality-of-life perception, even after a short, five-day intervention, confirming the effectiveness of CDT in postmastectomy lymphedema [34].

### 7.1. Manual Lymphatic Drainage

Manual lymphatic drainage (MLD) is a technique that is part of complex decongestive therapy, developed by Wiwarter in 1962 and later popularized by Vodder. The technique has been refined by Foldi, Leduc, and more recently by the Godoy and Godoy team. MLD is a specialized method that aims to activate the lymphatic system through a series of slow and rhythmic manual manipulations, performed with previous knowledge of the lymphatic anatomy. MLD increases the contractile activity of the lymphangions helping to propel the lymph to be reabsorbed by healthy lymphatic vessel [41]. The maneuvers used in MLD are distinct from conventional massage and are performed in a specific order, slowly and rhythmically, since the main objective will be to stimulate the lymphatic system to stimulate the lymphatic system and direct lymph to be reabsorbed to by healthy pathways for subsequent elimination [15,41].

### 7.2. Vodder Method

Emil Vodder is considered a pioneer of MLD [3]. Despite limited interest in the lymphatic system during his time, Vodder applied and refined the technique in patients with sinusitis, reporting favorable outcomes. The primary objective of his method is to activate lymphatic circulation and promote interstitial fluid clearance.

Vodder’s technique is based on four characteristic manipulations:

1. Fixed circles: fingers (excluding the thumb) are placed on the skin surface without pressure, performing a circular movement that gradually stretches the skin.
2. Pumping: the hand is positioned perpendicular to the treated area, with full palmar contact, applying a forward thrust.
3. Scoop (“Giving”): the hand is placed crosswise on the treated area, and with joints extended, a turning and pushing motion is performed.
4. Rotational: the hand is laid flat with the thumb at a 90° angle; a gentle twisting pressure is applied toward the little finger.

These movements are performed slowly, usually in a clockwise direction, following the anatomical lymphatic pathways. In upper-limb treatment, stimulation typically proceeds from proximal to distal—beginning in the subclavicular region, then the axillary nodes, and subsequently along the arm. Vodder did not emphasize complementing MLD with compression or exercise; this integration was later advanced by Földi, forming the basis of CDT.

### 7.3. Leduc’s Method

Albert Leduc expanded MLD by investigating the macro- and microcirculation of the lymphatic system [3]. He confirmed that lymphatic vessels respond to physical manipulations and classified MLD maneuvers into three categories:

1. Call maneuver: stimulates lymphatic collectors; pressure is applied progressively with shoulder abduction–adduction.
2. Ganglionic maneuver: mobilizes lymph nodes; circular movements combined with compression–decompression enhance nodal clearance.
3. Reabsorption maneuver: facilitates lymph evacuation from the edematous area; similar to the call maneuver but performed with different manual positioning.

For optimal results, these maneuvers must be performed slowly, respecting lymphatic physiology. Pressure should remain gentle, not exceeding 40 mmHg.

Leduc also documented collateral lymphatic pathways in cadaveric studies. Among more than 300 dissections, many collateral routes were identified in the posterior shoulder, potentially capable of draining the upper limb after mastectomy and lymphadenectomy [41,42]. Medina et al. [37] later confirmed these findings in breast cancer patients using indocyanine green lymphofluoroscopy: collateral pathways became visible after a 45-min MLD session, underscoring the importance of anatomical knowledge for effective treatment.

The clinical effectiveness of MLD remains debated. Huang et al. [35] conducted a meta-analysis of 10 randomized controlled trials (566 patients) comparing MLD with standard treatment, and found no significant benefit in preventing or reducing postmastectomy lymphedema, though methodological limitations were noted. Similarly, Liang et al. [36], in a review of 17 RCTs involving 1,911 patients, reported no significant isolated effect of MLD and called for further research due to insufficient evidence.

### 7.4. Godoy and Godoy Method:

In 1999, Godoy and Godoy introduced the Global Lymphatic Drainage technique [43], which integrates several therapeutic components:

1. Manual lymphatic drainage (MLD): gentle maneuvers designed to stimulate lymph uptake by capillaries for subsequent transport and evacuation.
2. Mechanical lymphatic therapy: use of mechanical devices to stimulate lymphatic flow.
3. Exercises and myolymphokinetic activities: the method distinguishes between daily activities (routine actions performed by the patient) and structured exercises (specific movements with repetitions and sets).
4. Cervical stimulation: gentle, superficial back-and-forth thumb movements in the supraclavicular fossa to stimulate lymphatic drainage.
5. Compression therapy: custom-made garments of gorgurão fabric, valued for their adjustability during the intensive treatment phase, accommodating changes in edema volume. However, this fabric is difficult to obtain in Mexico, as it must be imported from Brazil, increasing treatment costs.

The Godoy and Godoy approach also addresses psychological, nutritional, and social aspects, aiming for comprehensive patient well-being [15,43].

#### 7.4.1. Pneumatic Pressure Therapy

Pneumatic compression therapy applies intermittent external pressure through a pumping system on the affected limb. This pressure influences interstitial tissue, promoting lymph transport [41,43,44]. Its use is controversial: while some authors question its benefit and suggest that it may contribute to fibrosis [45], others report improved outcomes when combined with complex decongestive therapy (CDT). Florez et al. reviewed three clinical trials and found significant benefits when pneumatic compression was combined with MLD and compression garments [29].

#### 7.4.2. External Compression Devices

External compression is essential in lymphedema management. By increasing tissue pressure, it reduces edema, improves tissue elasticity, facilitates venous return, enhances fluid absorption, and supports lymphatic valve function [44,45]. Long-term management of lymphedema is rarely successful without compression.

Two primary modalities are used [46]:

- Multilayer bandaging: low-stretch bandages are applied from distal to proximal, creating a decreasing pressure gradient that promotes lymph flow (Laplace’s law). When combined with MLD, pressure gradients follow the same drainage pathway. Bandages are worn continuously during the intensive phase, then replaced by compression sleeves in the maintenance phase. Although effective, multilayer bandaging can be bulky and uncomfortable, requiring patient lifestyle adaptations. It is contraindicated in cases of infection, neuropathy, pain, sensory disorders, or vascular compromise. Application must be performed by trained professionals to ensure proper pressure gradients and avoid complications.
- Elastic compression sleeves: recommended for the maintenance phase. Sleeves are easier to use and better tolerated in daily life but less effective for rapid volume reduction compared with bandaging.

Several authors have evaluated the clinical effectiveness of external compressive devices. For instance, Damstra and Partsch [39] studied 36 postmastectomy patients with moderate-to-severe lymphedema, comparing low-pressure (20–30 mmHg) with high-pressure (44–58 mmHg) bandaging. After 24 h, the low-pressure group achieved a 9.2% volume reduction compared with 4.8% in the high-pressure group, while higher tolerance was reported at lower pressures. Nevertheless, compression decreased substantially after 2–24 h in both groups. Similarly, a Polish trial (2018) compared multilayer bandaging alone (n = 53) with CDT (n = 50) over 15 days in 103 patients. In both groups, significant improvement was observed, with no differences between them, which suggests that multilayer bandaging represents a cost-effective, time-saving, and essential element of CDT [32]. In addition, McNeely et al. [22] compared MLD plus multilayer bandaging versus bandaging alone in 50 women with breast-cancer-related lymphedema. In this study as well, both groups achieved significant volume reduction, with no differences between them.

Despite such findings, many lymphology experts emphasize that bandaging reduces only the liquid component of edema, not the protein-rich component within subcutaneous tissue, which can only be mobilized effectively by MLD. Thus, combining MLD with compression yields the best long-term outcomes [46].

### 7.5. Compression Sleeves

Compression sleeves are widely implemented to prevent edema recurrence following intensive treatment. Their main limitation is patient intolerance, as some individuals experience discomfort that reduces adherence [22]. For optimal effectiveness, sleeves should be custom-made, with correct sizing and compression levels tailored to the severity of lymphedema.

According to the protocol of the Physical Medicine Service at Valdeorras Hospital [47], class II compression is recommended for moderate lymphedema and class III compression for severe cases. Class I is not used, as it provides insufficient pressure. The protocol emphasizes tolerance and comfort: if the sleeve is uncomfortable, patients are more likely to abandon therapy. Compression sleeves are typically worn during the day and removed at night.

Evidence supports their clinical usefulness. Badger et al. [30] compared multilayer bandaging followed by compression sleeve use versus compression sleeves alone in 90 patients with lymphedema. The combined treatment achieved a 31% reduction in limb volume, compared with 15.8% in the sleeve-only group. Similarly, Ochalek, Gradalski, and Partsch [38] evaluated 54 postmastectomy patients: one group wore compression sleeves (15–20 mmHg) during the day, combined with education in hygiene, sleeve management, and physiotherapy; the control group did not use sleeves. At follow-up intervals of 1, 3, 6, 9, and 12 months, the sleeve group showed superior outcomes, leading the authors to conclude that compression sleeves are a safe and effective option for preventing postmastectomy lymphedema, particularly with continuous use between 3 and 12 months.

### 7.6. Lymphokinetic Exercises

Physical exercise is fundamental in the prevention and management of lymphedema in mastectomy patients. Reduced mobility may lead to muscle atrophy and diminished functional capacity, particularly in the operated upper limb. Muscle contraction during exercise acts as an extrinsic pump that facilitates lymphatic flow. For this reason, oncological physiotherapy should be an integral component of comprehensive breast cancer care. Exercise must be prescribed and supervised by trained professionals, tailored to each patient’s symptoms and clinical status, with the objective of preventing or reducing treatment-related side effects and improving quality of life [48,49,50].

Devoogdt et al. [24] studied the short- and long-term consequences of breast cancer treatment on shoulder mobility, lymphedema incidence, pain, and performance of activities of daily living (ADLs) in 77 patients undergoing radical or partial mastectomy with lymphadenectomy. At 3 months, 57.7% presented shoulder limitations and ADL impairments, decreasing to 31% at 3.4 years of follow-up. However, the incidence of lymphedema increased from 4% to 18%, mainly associated with shoulder pain and restricted mobility. The authors highlighted the importance of implementing structured exercise programs in the immediate postoperative period.

The timing of exercise initiation must align with surgical recovery. McNeely et al. [22] recommend initiating exercises one week post-surgery to minimize wound complications. Todd et al. [31] compared restricted versus unrestricted exercise programs in 116 patients, beginning on the second postoperative day. The unrestricted group, which performed full shoulder mobilization and rotation, reported 16 lymphedema cases after one year, suggesting that early unrestricted exercise may increase risk.

More recent evidence indicates that resistance training can be safe. In 2018, a controlled clinical trial followed 42 postmastectomy lymphedema patients for one year, comparing conventional CDT with CDT plus strength training using weights. Both groups improved mobility and strength, with no significant differences in limb volume, demonstrating that resistance exercise does not exacerbate lymphedema [33]. Similarly, Hasenoehrl et al. [51] performed a systematic review of 29 studies, concluding that resistance exercise does not negatively affect lymphedema and provides benefits by activating the muscle pump. They recommended clinical assessment prior to initiation and continuous supervision by trained professionals.

Exercise has therefore been shown to be both effective and safe for restoring shoulder mobility [52]. Programs should be individualized, but several guidelines provide practical recommendations. Organizations such as the Spanish Association Against Cancer (AECC), the Association of People with Lymphedema of Aragón (ADLAP), the American Cancer Society (ACS), and the Queen Sofia Hospital Care Guide for Mastectomized Women advise initiating exercise one week post-surgery, with medical authorization [26,52,53]. Initial routines should include 3–10 repetitions of each movement, repeated 2–3 times per day, avoiding pain or fatigue. Integration with respiratory exercises, especially diaphragmatic breathing, is emphasized.

Recommended activities include gentle active exercises without resistance, respecting the range of motion of the joints. Movements of the neck, shoulder girdle, and upper limb should be prioritized. Some guides suggest the use of simple objects (foam balls, broomsticks, towels, or books) to facilitate mobility. As recovery progresses, patients may gradually incorporate additional activities such as swimming, walking, stretching, low-intensity cardiovascular training, and light strengthening exercises, always under professional supervision [49,52,53].

### 7.7. Other Methods for the Treatment of Lymphedema

Several additional physical therapy techniques have been investigated for lymphedema management, although their effects appear less favorable than those of complex decongestive therapy (CDT). These include electrotherapy, low-level laser therapy, hydrotherapy, and kinesiotaping [22]. Current evidence indicates that kinesiotaping is not superior to CDT; however, it may help reduce pain or provide symptomatic relief during the early stages of lymphedema [15,54].

Pharmacological options, sometimes referred to as lymphopharmaceuticals, include diuretics, benzopyrenes, and antibiotics, aimed at stimulating lymphatic flow or preventing infections [17]. Their use should be restricted to carefully selected patients and prescribed only by specialized physicians. Calderón González et al. [55] conducted a literature review and found insufficient evidence to broadly recommend these agents, suggesting their role is limited to specific clinical contexts

### 7.8. Surgical Methods

Surgical treatment is generally reserved as an adjunct to CDT or as an alternative for patients with advanced lymphedema who do not respond adequately to conservative measures. The objectives of surgery are to reduce limb volume, improve functionality, and prevent recurrent infections such as cellulitis or lymphangitis. Best outcomes are observed when interventions are performed early, particularly in stages I and II, where lymphaticovenous anastomosis (LVA) followed by CDT has demonstrated effectiveness [56,57]. Surgery should only be performed by medical specialists with advanced training in lymphology.

Recent technological innovations have expanded surgical options. A pilot study demonstrated the feasibility of robot-assisted supermicrosurgical lymphatic anastomosis using the MUSA robotic system. In the first human trial, patients with breast-cancer-related lymphedema experienced reduced arm volume and improved quality of life three months after surgery. These findings confirm the feasibility and safety of robotic supermicrosurgery; however, further trials with larger samples and specialized training for microsurgeons are necessary to validate its long-term therapeutic potential [58].

Although surgical alternatives are promising, their implementation is limited by the need for highly trained professionals and the significant costs associated with these procedures.

## 8. Discussion

In Mexico, the limited level of knowledge of lymphedema prevention in breast cancer patients is reflected in the scarce coverage of this issue in normative documents related to breast cancer care. For example, the Mexican Official Standard NOM-041-SSA2-2011 states that all patients with breast cancer should “receive adequate rehabilitation according to the case, with physiotherapy, the use of breast prostheses, breast reconstruction, or lymphedema treatment,” yet it does not specify how physiotherapy should be administered or define treatment conditions. Similarly, the Clinical Practice Guide of the Mexican Social Security Institute recommends informing and educating patients on prevention and management of lymphedema, but provides limited operational guidance. The Mexican Council on Breast Cancer Diagnosis and Treatment (CMDTCM) acknowledges that rehabilitation remains poorly understood, resulting in a higher incidence of lymphedema than could be expected with proper preventive measures. Among official documents, the CMDTCM is one of the few to include exercise recommendations for postmastectomy patients [59].

Current statistics highlight the urgent need for effective prevention and treatment strategies. In Mexico, approximately 42% of breast cancer cases are diagnosed in advanced stages, requiring aggressive treatment such as mastectomy with lymphadenectomy and radiotherapy—both strong risk factors for lymphedema. About 30% of patients undergoing surgery will develop lymphedema, most often within the first 12 months postoperatively [16,59]. National epidemiological data remain scarce, but available studies indicate that breast cancer is the primary cause of cancer-associated lymphedema in the country. Furthermore, many patients present with obesity, overweight, and low physical activity, complicating management and often leading to advanced-stage diagnoses (stage II or III) associated with irreversible disabilities. Disseminating information in public health services and promoting preventive measures and healthy lifestyle habits are therefore essential for preserving quality of life.

Lymphedema is a chronic and progressive condition, and patients must be informed about its potential as a sequela of oncological treatment [16,17,59]. Prevention plays a fundamental role. Studies on secondary prevention programs after mastectomy have consistently shown improved outcomes when patients are educated about early signs and symptoms, trained in preventive measures, and supervised by specialized health personnel [21,23,25,28]. Ideally, surveillance programs, such as the model proposed by Stout et al. [19], should be integrated into the Mexican health system, covering preoperative, immediate postoperative, and follow-up stages, to enable early detection and timely intervention.

Treatment should be administered by health professionals trained in lymphology, with theoretical and practical mastery of manual lymphatic drainage (MLD), multilayer bandaging, compression therapy, and therapeutic exercise. These professionals must also develop decision-making skills to identify risk factors, prevent complications, and select optimal treatment strategies. Historically, Mexico has lacked specialization in lymphology or oncology for physiotherapists; diploma courses, first offered in 2007, remain the highest level of training available. Although associations and universities have recently expanded training opportunities, undergraduate programs in physical therapy and medicine still dedicate little curricular attention to lymphedema.

The international literature identifies complex decongestive therapy (CDT) as the first-line treatment, integrating MLD, compression therapy, lymphokinetic exercises, and self-care measures [17]. Although CDT is endorsed by major associations such as the ISL, BreastCancer.org, and the Mexican Consensus on Breast Cancer, some studies have evaluated isolated components. For example, Florez et al. [29] showed that multilayer bandaging alone produced significant volume reduction, with lower costs and reduced treatment time. Similarly, McNeely et al. [22] demonstrated volume decreases with multilayer bandaging, regardless of whether MLD was added.

Nevertheless, external compression remains indispensable for successful treatment [16,17,26,50,55]. Its correct application requires trained personnel and quality materials, which may generate additional costs for patients. Damstra and Partsch compared low- versus high-pressure multilayer bandaging and underscored the importance of accurate pressure measurement and maintenance [39].

Manual lymphatic drainage, first described in 1962, continues to evolve. Modern imaging techniques such as lymphography and indocyanine green lymphofluoroscopy have advanced understanding of lymphatic flow, enabling more precise and individualized application of MLD. These innovations may improve clinical reasoning, particularly in breast cancer patients with disrupted lymphatic pathways [8,9,10,11]. However, systematic reviews by Huang et al. [35] and Liang et al. [36] concluded that MLD is ineffective as an isolated treatment due to methodological inconsistencies and insufficient evidence. Despite this, MLD remains valuable when integrated into CDT, as no comparative studies to date have determined which method (Vodder, Leduc, or Godoy) is superior.

Therapeutic exercise has emerged as a safe and effective intervention, particularly when initiated in the immediate postoperative period. McNeely et al. [22] and Todd et al. [31] recommended initial restrictions during the first week post-surgery to minimize complications. Later studies, such as those by Hasenoehrl et al. [51] and Luz et al. [33], dispelled concerns that resistance training might exacerbate lymphedema, instead showing that it improves strength and does not increase limb volume. Guidelines from international associations reinforce that exercise programs should be supervised, individualized, and combined with self-care measures, which remain essential at all stages of lymphedema management [26,52,53].

## 9. Conclusions

Lymphedema remains a significant and often underestimated sequela of breast cancer treatment in Mexico. Despite its impact on functionality and quality of life, national guidelines and clinical practice protocols provide only limited and non-specific recommendations, resulting in delayed detection and management. The high proportion of breast cancer cases diagnosed at advanced stages, combined with risk factors such as obesity and sedentary lifestyles, further increases the incidence and severity of lymphedema in Mexican patients.

Evidence from the international literature consistently identifies complex decongestive therapy (CDT) as the gold standard for lymphedema management, combining manual lymphatic drainage, compression therapy, exercise, and self-care strategies. Although some studies have reported benefits of isolated components—particularly compression—multimodal approaches remain superior in terms of long-term outcomes. Early physiotherapy interventions, structured exercise programs, and patient education have also demonstrated efficacy in reducing incidence, controlling progression, and improving functionality.

In Mexico, strengthening the response to lymphedema requires a multilevel strategy that integrates prevention, early diagnosis, and management guidelines into national oncology standards; trains specialized professionals in lymphology and rehabilitation to deliver evidence-based interventions such as CDT, manual lymphatic drainage, multilayer bandaging, and tailored exercise programs; incorporates comprehensive lymphedema education into undergraduate and postgraduate curricula in physical therapy and medicine; and systematically educates patients on risk factors, early signs, and self-care strategies to promote adherence and active participation in their own care.

Addressing these gaps will not only reduce the burden of lymphedema but also preserve the functional capacity, autonomy, and quality of life of breast cancer survivors in Mexico.

## Figures and Tables

**Table 1:** Summary of the main articles reviewed in the paper on the prevention of lymphedema.

Author (Year)	Objective	Subjects	Level of Prevention	Conclusions
Torres (2010) [18]	To evaluate the efficacy of an early physiotherapy program for the prevention of lymphedema in women undergoing breast surgery with lymphadenectomy.	116 women	Secondary prevention	Early physiotherapy could be an effective measure to prevent lymphedema in patients treated with breast surgery with lymphadenectomy, in the first year after surgery.
Stout et al. (2012) [19]	To propose a surveillance model for post-mastectomy patients during the preoperative, immediate postoperative, and survival periods to identify disabilities and limitations associated with breast cancer treatment.		Secondary prevention	The implementation of the care model allows planning the functional rehabilitation of the patient, preventing sequelae.
Forner (2003) [20]	To develop and apply a questionnaire to analyze the quality of information that patients receive after surgery in relation to lymphedema and the consequences of breast cancer.	65 patients	Secondary prevention	Few post-mastectomy patients receive information about the risk of developing lymphedema and its prevention, the content of which is poor and nonspecific.
Fenglian et al. (2020) [21]	To establish a scoring system to predict the risk of breast-cancer-related lymphedema.	533 patients	Secondary prevention	The predictive efficiency and accuracy of the scoring system were acceptable, and the system could be used to predict and evaluate groups at high risk of breast-cancer-related lymphedema.
McNeely et al. (2010) [22].	To collect information and compare findings regarding the effectiveness of interventions to prevent, minimize, or improve upper limb dysfunctions due to breast cancer treatment.	24 studies	Secondary prevention	Upper limb exercise is helpful in regaining upper limb movement after breast cancer surgery. Structured exercise programs during the first few weeks after surgery are beneficial for regaining movement.
Boccardo, et al. (2009) [23]	To evaluate the effectiveness of teaching preventive measures for the management of lymphedema.	55 patients	Secondary prevention	The population was divided into two groups. The group that received teaching on care, reduction in risk factors, and timely diagnosis of lymphedema had a lower incidence of cases.
Devoogdt, et al. (2011) [24].	To evaluate the short- and long-term effects of cancer treatment in terms of the impact they have on shoulder mobility and the appearance of lymphedema.	77 patients	Secondary prevention	Over time, a significant number of the population continue to suffer from mobility problems and lymphedema has increased detrimentally.
Josephine (2019) [25]	To evaluate a protocol for the prevention of lymphedema and quality of life in patients with mastectomy.	120 patients	Secondary prevention	The comparison of the quality of life between the study group and the control group was statistically significant for the improvement in quality of life, preventing the appearance of lymphedema.

**Table 2:** Summary of the main articles reviewed in the papers on main physiotherapy techniques applied to the treatment of lymphedema.

Author (Year)	Applied Technique	Objective	Theoretical Basis	Application and Results
McNeely, et al., (2010) [22]	Multilayer compression bandage Manual lymphatic drainage	To evaluate the efficacy of multilayer compression bandaging with and without manual lymphatic drainage.	Volume reduction by means of compression bandaging acts exclusively on the fluid and not on the proteins concentrated in the subcutaneous tissue, a function that is important to perform by means of DLM.	Lymphedema decreased in both cases without significant differences.
Badger, et al. (2000) [30]	Compression therapy: Compression sleeve Multilayer bandage.	To compare the effect of multilayer bandaging as an initial phase of lymphedema treatment followed by a compression sleeve, vs. the exclusive use of a compression sleeve.	Multilayer bandaging as the initial phase of treatment for patients with lymphedema, followed by stockings, achieves a greater and more sustained reduction in limb volume than sleeves alone.	In the combined treatment group, there was a two-fold reduction in volume compared to the group that used the compression sleeve alone.
Todd (2008) [31]	Therapeutic exercise.	To evaluate the benefits of shoulder mobilization in the immediate postoperative period.	It is preferable to perform restricted mobility exercises within the immediate postoperative period to avoid complications associated with the recovery process following surgery.	Patients who performed restricted exercise during the first two weeks after surgery had fewer complications.
Zasadzka et al. (2018) [32]	Complex decongestive therapy Multilayer compression bandage.	To compare the effectiveness of using multilayer bandage and complex decongestive therapy.	Multilayer compression bandage is an element of CDT, its isolated use saves treatment costs and therapy time, with favorable volume reduction benefits.	Multilayer bandaging is essential for TDC, which alone can provide the desired results such as volume reduction.
Luz et al. (2018) [33]	Complex decongestive therapy	To compare a complex decongestive therapy protocol alone or combined with muscle strength training in patients with lymphedema.	Studies suggest that patients with lymphedema should not perform high-intensity exercise. However, other studies have shown that properly guided strength exercises can help reduce lymphedema.	There was no difference in limb volume. Patients with lymphedema can safely perform strengthening exercises without the risk of increasing the volume of the edema-affected upper limbs.
Cruz-Ramos et al. (2017) [34]	Complex decongestive therapy	To evaluate the effects of CDT for reducing limb volume in lymphedema.	Intensive phase treatment was carried out for five days, covering all the elements of CDT.	CDT helps to reduce the volume of the limb with lymphedema, despite having a short intervention period.
Huang et al. (2013) [35]	Manual lymphatic drainage	To conduct a systematic review of randomized controlled trials to assess the efficacy of MLD in preventing and treating breast-cancer-related lymphedema.	DLM, in conjunction with skin care, exercise, and compression bandaging, provides optimal management of lymphedema.	Current evidence does not support the use of MLD to prevent or treat lymphedema. However, methodological inconsistencies were found in the studies analyzed.
Liang et al. (2020) [36]	Manual lymphatic drainage	To summarize the current evidence to assess the effectiveness of MLD in preventing and treating lymphedema in patients after breast cancer surgery.	Studies have shown that DLM has a beneficial effect on lymphedema related to breast cancer surgery.	DLM may not have any isolated effect for the prevention and treatment of lymphedema. Further research should be carried out as there was no adequate level of evidence to prove whether it is effective or not.
Medina et al. (2019) [37]	Manual lymphatic drainage	To identify and describe the presence of collateral drainage areas and pathways, using lymphofluoroscopy with indocyanine green, in patients with secondary lymphedema of the upper limbs after breast cancer, treated with DLM.	Manual lymphatic drainage appears to stimulate lymphatic contraction, helping the development of secondary pathways and stimulating the appearance of collateral pathways that could function as the main drainage pathways of the limb in cases of lymphedema.	Additional pathways were observed after MLD, the use of lymphofluoroscopy techniques with indocyanine green is a useful tool to know the effective drainage pathways in lymphedema secondary to cancer treatment.
Ochalek et al. (2017) [38]	Compression sleeve	To evaluate the role of compression sleeves in the prevention and management of post-mastectomy lymphedema.	The use of compression media facilitates lymph flow. The compression sleeve is an easily accessible device for the patient, and its daily use facilitates lymph flow.	The use of compression sleeves is a safe and effective option to prevent post-mastectomy lymphedema, especially between 3 and 12 months after use.
Damstra and Partsch (2019) [39]	Multilayer bandage	To determine whether there is a difference in results between low- and high-pressure multilayer bandages, in relation to volume reduction and patient tolerance to the bandage.	Using low pressure generates better adherence to the treatment since it is better tolerated by the patient.	The low pressure group had better results in terms of volume reduction and better pressure tolerance.
